# A systematic review of interventions to improve postpartum retention of women in PMTCT and ART care

**DOI:** 10.7448/IAS.19.1.20679

**Published:** 2016-04-25

**Authors:** Pascal Geldsetzer, H Manisha N Yapa, Maria Vaikath, Osondu Ogbuoji, Matthew P Fox, Shaffiq M Essajee, Eyerusalem K Negussie, Till Bärnighausen

**Affiliations:** 1 Department of Global Health and Population, Harvard T.H. Chan School of Public Health, Boston, MA, USA; 2 Africa Centre for Population Health, University of KwaZulu Natal, Mtubatuba, South Africa; 3 Center for Global Health & Development, Boston University, Boston, MA, USA; 4 HIV/AIDS Department, World Health Organization, Geneva, Switzerland

**Keywords:** PMTCT, retention, Option B+, postpartum, HIV, antiretroviral therapy, loss to follow-up

## Abstract

**Introduction:**

The World Health Organization recommends lifelong antiretroviral therapy (ART) for all pregnant and breastfeeding women living with HIV. Effective transitioning from maternal and child health to ART services, and long-term retention in ART care postpartum is crucial to the successful implementation of lifelong ART for pregnant women. This systematic review aims to determine which interventions improve (1) retention within prevention of mother-to-child HIV transmission (PMTCT) programmes after birth, (2) transitioning from PMTCT to general ART programmes in the postpartum period, and (3) retention of postpartum women in general ART programmes.

**Methods:**

We searched Medline, Embase, ISI Web of Knowledge, the regional World Health Organization databases and conference abstracts for data published between 2002 and 2015. The quality of all included studies was assessed using the GRADE criteria.

**Results and Discussion:**

After screening 8324 records, we identified ten studies for inclusion in this review, all of which were from sub-Saharan Africa except for one from the United Kingdom. Two randomized trials found that phone calls and/or text messages improved early (six to ten weeks) postpartum retention in PMTCT. One cluster-randomized trial and three cohort studies found an inconsistent impact of different levels of integration between antenatal care/PMTCT and ART care on postpartum retention. The inconsistent results of the four identified studies on care integration are likely due to low study quality, and heterogeneity in intervention design and outcome measures. Several randomized trials on postpartum retention in HIV care are currently under way.

**Conclusions:**

Overall, the evidence base for interventions to improve postpartum retention in HIV care is weak. Nevertheless, there is some evidence that phone-based interventions can improve retention in PMTCT in the first one to three months postpartum.

## Introduction

The human immunodeficiency virus (HIV) can be transmitted from the mother to her child during pregnancy and birth through blood, or postnatally through breast milk. With mounting evidence from randomized trials of the safety and efficacy of postnatal maternal antiretroviral drugs in preventing HIV transmission through breast milk [1–6], the finding that antiretroviral therapy (ART) drastically reduces the risk of sexual transmission [7,8], and the benefits of ART to the patient's health even at high CD4-cell counts [9], the World Health Organization's (WHO) prevention of mother-to-child HIV transmission (PMTCT) guidelines have gradually moved to recommending longer periods of maternal antiretroviral drugs postpartum. In its latest 2015 early-release guidelines, the WHO recommends providing lifelong ART to all pregnant and breastfeeding women living with HIV regardless of CD4-cell count or clinical stage, which has also been termed Option B+ in earlier guidelines [10]. Countries have moved fast to implement this option, with 18 of 22 priority countries having adopted or scaled-up Option B+ to date [11]. Five countries (Malawi, Tanzania, Uganda, Lesotho, and Ethiopia) have nationally implemented lifelong ART for all pregnant and breastfeeding women living with HIV, and an additional eight countries have started to scale up this treatment option.

Pregnant women who test HIV-positive in antenatal care (ANC) are generally initiated on antiretroviral drugs at the antenatal clinic, or after referral from ANC to ART care settings. After a series of prenatal PMTCT visits, the mother is usually expected to attend a number of further postnatal PMTCT visits for both herself and her child. The existing literature shows that a large percentage of women are lost along this cascade [12]. While the evidence on postpartum retention is sparse, depending on the setting and follow-up period, approximately 25–50% of women on ART at delivery may be lost from care during the postpartum period [13–15]. This high attrition rate is problematic due to the increased risks of HIV-related morbidity and mortality, HIV transmission, and the development of drug-resistant HIV strains [16,17].

While the move to Option B+ simplifies the guidelines for ART by recommending that all pregnant women, regardless of CD4 count or clinical stage, begin lifelong ART, it introduces a new step in the care cascade between PMTCT or maternal and child health (MCH) care services, and long-term ART services. Finding ways to transition women initiating HIV treatment in the antepartum period to lifelong ART postpartum, and retaining them in ART care, will thus be critical to reducing both vertical and sexual HIV transmission, and to improving the mother's health. More broadly, studying these questions is likely to yield lessons that are pertinent to a wide variety of healthcare settings. Other than providing evidence on how retention in long-term care settings can be improved, such studies may also help to answer how a previously stand-alone programme can be effectively integrated into a broader care cascade. In addition, evidence on how retention of postpartum women in HIV care can be increased is likely to apply to other care settings, in which an important motivational factor for the patient to attend care (in this case, reducing the risk of infecting the fetus/newborn with HIV) has ceased to exist.

Focusing on HIV-positive women between birth and five years postpartum, we conducted a systematic review to assess the evidence for interventions that aim to improve (1) retention within PMTCT programmes, (2) transitioning from PMTCT to general ART programmes, and (3) retention in general ART programmes.

## Methods

This review was commissioned by the WHO to inform the operational section of the 2015 update of the WHO's consolidated guidelines on the use of antiretroviral drugs. A review protocol exists and can be requested from the authors.

### Search strategy

We conducted searches for data published since January 1, 2002, in the following databases: PubMed (searched on April 18, 2015), ISI Web of Knowledge (searched on May 1st 2015), Embase (searched on May 7, 2015), WHO regional indexes (AIM, LILACS, IMEMR, IMSEAR, WPRIM; all searched on May 14, 2015, via the Global Health Library), and the Cochrane Central Register of Controlled Trials (searched on May 17, 2015). We also searched conference abstracts for the Conference of the International AIDS Society (IAS and AIDS) from 2002 to 2014, and the Conference on Retroviruses and Opportunistic Infections (CROI) in 2014 and 2015. The databases were searched using key words and medical subject headings (MESH) for HIV, PMTCT, retention in care, loss to follow-up, transition, linkage, and pregnancy. The search terms for each database are shown in additional file 1. We screened titles and abstracts for relevance, and then analyzed the full-text versions of potentially relevant articles using the following inclusion criteria: (1) the study population is pregnant or postpartum women (up to a maximum of five years after delivery) living with HIV, (2) the article presents primary quantitative data on the transition from ANC or PMTCT to ART in the postpartum period, or data on retention in PMTCT or ART care of postpartum women living with HIV, and (3) the study evaluates an intervention. The database searches, and analyses of abstracts and articles’ full-text versions were not conducted in duplicate. No restrictions were placed on study design, sample size, or publication type. We also did not restrict articles by publication language although the search strategy was only run in English. Finally, the reference lists of all included studies, and relevant review articles and commentaries were screened for additional references. We also contacted the corresponding author of all included studies and relevant ongoing studies, for which we found a published study protocol (see Table 10), to inquire about unpublished data and manuscripts.

### Data extraction

The following information was extracted from each included article: author, year of publication, period of data collection, study design, country, study population, unit of randomization (for randomized studies only), sample size by study arm, outcome measure(s), and study results.

### Assessment of study quality

We assessed the quality of each included study using the criteria of the Grades of Recommendation, Assessment, Development and Evaluation (GRADE) Working Group [18]. As such, each study was graded as high, moderate, low, or very low quality of evidence. We decided to grade quality by study rather than outcome because of the wide heterogeneity in outcome measures and intervention designs of the included studies. We did not undertake an assessment of risk of bias across studies (e.g., publication bias).

### Analysis of extracted data

We extracted or calculated, if possible, the proportion of women who were successfully transitioned to, or retained in ART. We also determined the relative risk (RR) or odds ratio (OR) with 95% confidence intervals (CIs) comparing the rate of transitioning and/or retention between the intervention and control group. Due to the large degree of heterogeneity between study designs, interventions, outcome measures, and the reporting of outcomes, we decided that a meta-analysis was inappropriate.

## Results and discussion

**Figure 1:**
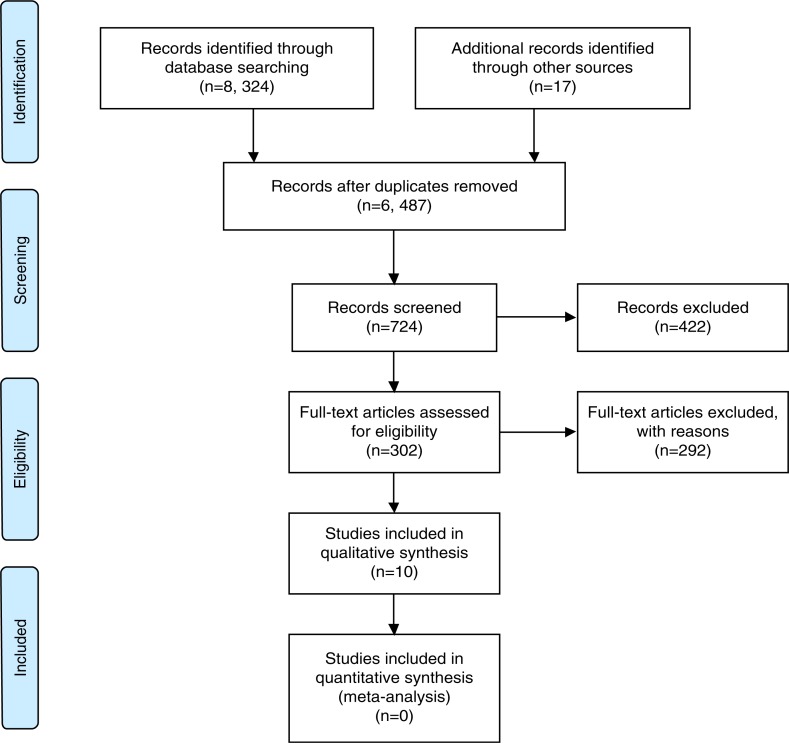
Prisma flow diagram summarizing the literature search.

The search of the databases retrieved 8324 records with an additional 17 records identified through searching the reference lists of included articles and relevant reviews, and contacting authors (Figure 1). After removing duplicates, screening abstracts, and full-text reviews, we identified 10 studies that met our inclusion criteria.

### Characteristics of included studies

Nine of ten studies included in this review were carried out in sub-Saharan Africa (one study took place in the United Kingdom), seven of which were conducted in East Africa and two in South Africa (Tables 1–3). All studies were published between 2010 and 2015. Six studies were observational studies and four randomized trials, of which one was a cluster-randomized trial. Three of the studies evaluated interventions that employed text messages and/or phone calls to improve retention, and four studies evaluated the effect of different levels of integration between ANC/PMTCT and ART care on retention.

**Table 1:** Characteristics of studies that evaluated interventions using phone calls and/or text messages

Author and year					Sample size				
							
	Country	Study period	Study design	Study population	*Intervention*	*Control*	Intervention	Control group	Outcome measure
Odeny *et al*. 2014 [19]	Kenya	April 2012 to March 2013	Individually randomized trial	HIV+ pregnant women enrolled in PMTCT at any of five health facilities, between 28 weeks gestation and delivery	195 women	193 women	14 text messages (eight during pregnancy, six postpartum)+weekly calls from 38 weeks’ gestation until delivery	Standard care+contacting women by phone or in person if did not attend a PMTCT visit	Attendance of a PMTCT or postnatal clinic appointment in the first eight weeks postpartum
Kebaya *et al*. 2014 [20]	Kenya	Not stated	Individually randomized trial	HIV+ mothers (and their infant) who delivered at one of three health facilities	75 MIPs	75 MIPs	Biweekly phone call reminders about PMTCT in the first 10 weeks postpartum	Standard care (no phone calls)	Proportion of women who attended a Maternal and Child Health clinic appointment at six and ten weeks postpartum
Schwartz *et al*. 2015 [21]	South Africa	May 2013 to July 2013	Pre/post cohort study	HIV+ pregnant women ≥36 weeks gestation attending antenatal care and receiving ART through the Option B+ programme	50 women	50 women	Text messages and phone calls through six weeks postpartum	HIV+ pregnant women ≥36 weeks gestation attending antenatal care and receiving ART through the Option B+ programme	Retention^a^ at 12-months postpartum

HIV+=HIV-positive; ART=antiretroviral therapy; PMTCT=prevention of mother-to-child HIV transmission; MIP=mother-infant pair.

a Retention was defined as not having missed the last appointment to pick up antiretroviral drugs by more than six weeks, or having transferred out to another healthcare facility.

**Table 2:** Characteristics of studies evaluating interventions that integrated care

Author and year		Study period			Sample size				
						
	Country		Study design	Study population	*Intervention*	*Control*	Intervention	Control group	Outcome measure
Turan *et al*. 2015 [22]	Kenya	June 2009 to March 2012	Cluster-randomized trial	HIV+ pregnant women not previously enrolled in HIV care	Six healthcare facilities (596 women)	Six healthcare facilities (603 women)	Integrated care at ANC (ANC+HIV care and treatment in the same clinic until 18 months post-partum)	Standard care (ANC+referral for HIV care and treatment at the same facility but a different clinic)	(1) Enrolment in HIV care within 12 months after testing HIV+ in ANC (2) Time to women's enrolment in HIV care (3) Proportion of eligible women who initiated ART within 12 months of testing HIV+ in ANC (4) Proportion of women with two or more HIV care follow-up visits in the first six months after testing HIV+ in ANC
van Lettow *et al*. 2014 [23]	Malawi	January 2012 to June 2013	Cohort study	HIV+ pregnant women who started ART under Option B+	136 facilities (73 in Model A, 36 in Model B, 18 in Model C, 9 in Model D)	N/A	Compares facilities falling into four different levels of ANC and ART services integration^a^	N/A	(1) Proportion of HIV+ women (not already on ART) initiated on ART during pregnancy (2) Retention^b^ at 12 months after ART initiation (3) Relative likelihood of high retention^b^ at a facility in each model of care at six months after ART initiation
Weigel *et al*. 2012 [24]	Malawi	July 2006 to October 2010	Pre/post cohort study	HIV+ pregnant women with CD4 < 250 and not on ART at first ANC visit	133 women	53 women	Series of interventions over three years to enhance linkage between ANC and ART^c^	HIV+ pregnant women with CD4 < 250 and not on ART at first ANC visit prior to/at beginning of linkage-enhancing interventions	Retention^d^ at six months after ART initiation
Stinson *et al*. 2010 [25]	South Africa	January 2005 to December 2005	Cohort study	HIV+ pregnant women with CD4 < 200 and not on ART at first ANC visit	4 clinics (516 women)	N/A	Three models of care for ANC to ART linkage^e^	N/A	(1) Initiation of ART before delivery (2) Initiation of ART between delivery and two years postpartum (3) Initiation of ART at any time before two years postpartum

HIV+=HIV-positive; ART=antiretroviral therapy; PMTCT=prevention of mother-to-child HIV transmission; ANC=antenatal care; N/A=not applicable; CD4=cluster of differentiation 4 cell count; MIP=mother-infant pair.

a The four different levels of ANC and ART integration are: (1) women are initiated and followed on ART at ANC until birth (Model A); (2) women receive only the first dose of ART at ANC, and are referred to the ART clinic for follow-up (Model B); (3) women are referred from ANC to the ART clinic for initiation and follow-up (Model C); (4) facilities not providing ANC, but serving as ART referral sites (Model D).

b Retention was defined as (1) not expected to have run out of ARVs for two or more months (based on the number of tablets given at the last clinic visit), or (2) known to have transferred out, stopped or died.

c The interventions consisted of provider-initiated CD4-testing (July 2006), introduction of a paper-based referral system between ANC and ART services (July 2006), provision of transport between ANC and ART clinic (September 2006), opening of a new HIV clinic in walking distance from ANC facility (December 2006), designation of a PMTCT link person (December 2006), introduction of a standardized referral letter May 2007), revoking of necessity for presence of guardian to initiate ART (May 2007), additional counselling session at ART facility (May 2007), leaflets, posters and signposts aimed at informing women how to get from the ANC to the ART facility (March 2008), introduction of an electronic medical system including a unique hospital patient identification number (December 2008), and relocation of ANC facility (October 2009).

d Retention was defined as being alive and on ART, or having transferred to another ART facility.

e “Integrated model”: ANC and ART offered in the same clinic; “proximal model”: referral from ANC to an ART service in a separate building but on the same premises; “distal model”: referral from ANC to ART services in a 5-km radius (accessible by foot or public transport from the ANC clinic).

**Table 3:** Characteristics of other included studies

Author and year					Sample size				
							
	Country	Study period	Study design	Study population	*Intervention*	*Control*	Intervention	Control group	Outcome measure
Kiweewa *et al*. 2013 [26]	Uganda	May 2007 to September 2009	Individually randomized trial	ART-naïve women with CD4 < 200 referred from PMTCT programme (ante- or post-partum) for ART initiation for life at a national referral hospital	45 women	40 women	Less frequent visits mostly managed by an ART nurse and peer counsellor^a^	Standard care (monthly ART care by a doctor+routine counselling by a nurse counsellor at each visit)	Attendance of all scheduled visits in the first 12 months after ART initiation
Mushamiri *et al*. 2015 [27]	Kenya	October 2010 to January 2013	Cohort study	HIV+ pregnant women who attended ANC at one of eight facilities	Area 1: 84 women Area 2: 45 women	Area 3: 47 women	Area 1: CHW services^b^ Area 2: CHW services+text message reminders to CHWs^c^	Area 3: No CHW services	Proportion of women who attended six or more baby follow-up visits in the first 18 months postpartum
Williams *et al*. 2014 [28]	United Kingdom	June 2008 to December 2011	Pre/post cohort study	HIV+ pregnant women	26 women	25 women	Financial support for formula feeding provided at ART clinic appointments	Standard care (no financial support)	(1) Proportion of women who discontinued ART after delivery despite CD4 < 350 (2) Proportion of women on ART with viral load ≥200 at any ART visit in first 12 months postpartum (3) Mean number of ART appointments not attended in the first 12 months postpartum

HIV+=HIV-positive; ART=antiretroviral therapy; CHW=community health worker; PMTCT=prevention of mother-to-child HIV transmission; ANC=antenatal care; CD4=cluster of differentiation 4 cell count.

a Participants in the intervention arm were seen at baseline, week 2, and months 1, 2, 3, 6, 9 and 12, of which the baseline visit and the visits at months 2 and 12 were managed by a doctor. Participants in the control arm where seen at baseline, weeks 2 and 4, and monthly thereafter (all visits were managed by a doctor).

b CHWs were responsible for (1) identifying newly pregnant women through household visits; (2) referring newly pregnant women to ANC, (3) reminding women of an upcoming ANC, PMTCT, or baby follow-up appointment, (4) visiting women at home who have missed an ANC, PMTCT, or baby-follow-up appointment, and (5) visiting women at home two weeks before the due date to discuss the birth plan. In Area 2, these activities were overseen by the Millennium Villages Project and closely monitored. In Area 1, they were overseen by the government or other non-governmental organizations.

c CHWs registered women in an automatic text message system at the first ANC/PMTCT visit. The system sent an automatic reminder to the CHWs asking them to (1) remind women of an upcoming ANC, PMTCT, or baby follow-up appointment, (2) visit a woman at home who had missed an ANC, PMTCT, or baby follow-up appointment, and (3) visit a woman to discuss her birth plans two weeks prior to her due date.

### Quality assessment

Four of the included studies were of high or moderate quality, and six of very low quality (Tables 4–6). We downgraded all observational studies in this review to very low quality. Reasons for the downgrade were a high risk of bias and imprecision of results (i.e., wide confidence intervals). The justification for each study's quality grading is shown in Tables 4–6.

**Table 4:** Quality of included studies according to the GRADE criteria – phone calls and/or text messages

Study	Risk of bias	Inconsistency	Indirectness	Imprecision	Quality	Justification
Odeny *et al*., Kenya, randomized trial [19]	No	No	Serious	No	Moderate ⊕⊕⊕⊖	Randomized trial downgraded due to indirectness from needing to have access to a mobile phone to be eligible for enrolment
Kebaya *et al*., Kenya, randomized trial [20]	No	No	Serious	No	Moderate ⊕⊕⊕⊖	Randomized trial downgraded due to indirectness from needing to have access to a mobile phone to be eligible for enrolment
Schwartz *et al*., South Africa, cohort study [21]	Serious	No	No	Serious	Very low ⊕⊖⊖⊖	Observational study downgraded due to (1) prospective data collection for intervention vs. retrospective data collection for control group, and (2) a wide CI

CI=confidence interval.

**Table 5:** Quality of included studies according to the GRADE criteria – integration of care

Study	Risk of bias	Inconsistency	Indirectness	Imprecision	Quality	Justification
Turan *et al*., Kenya, randomized trial [22]	Serious	No	No	Serious	Moderate ⊕⊕⊕⊖	Randomized trial downgraded due to (1) risk of bias from incomplete medical records, and (2) a wide CI in the retention outcome
van Lettow *et al*., Malawi, cohort study [23]	Serious	No	No	Serious	Very low ⊕⊖⊖⊖	Observational study downgraded due to (1) risk of confounding from factors that influenced facilities’ adoption of a certain delivery model, and (2) wide CIs
Weigel *et al*., Malawi, cohort study [24]	Very serious	No	Very serious	No	Very low ⊕⊖⊖⊖	Observational study downgraded due to (1) risk of bias from time trends over the long intervention period, and (2) the large number of sequential interventions implemented
Stinson *et al*., South Africa, cohort study [25]	Very serious	No	No	No	Very low ⊕⊖⊖⊖	Observational study downgraded due to risk of bias from inter-clinic variability as only four facilities were chosen for three models of care

CI=confidence interval.

**Table 6:** Quality of included studies according to the GRADE criteria – other interventions

Study	Risk of bias	Inconsistency	Indirectness	Imprecision	Quality	Justification
Kiweewa *et al*., Uganda, randomized trial [26]	No	No	No	No	High ⊕⊕⊕⊕	Randomized trial
Mushamiri *et al*., Kenya, cohort study [27]	Serious	No	No	No	Very low ⊕⊖⊖⊖	Observational study downgraded because women in the study groups were not matched (or analyzed) based on socio-demographic or clinical characteristics
Williams *et al*., UK, cohort study [28]	Serious	No	No	Serious	Very low ⊕⊖⊖⊖	Observational study downgraded due to (1) risk of bias from time trends, and (2) wide CIs

CI=confidence interval.

### Outcomes with relevant summary measures

Two randomized studies, both of moderate quality according to GRADE criteria, found that the use of text messaging and/or phone calls was associated with improved clinic attendance postpartum (Table 7). Odeny *et al*. found that two-way text messaging was associated with an increase in the percentage of patients who attended a postpartum visit within eight weeks of delivery (RR: 1.66, 95% CI: 1.02–2.70) [19], and Kebaya *et al*. found that biweekly phone calls during the first 10 weeks postpartum were associated with a higher proportion of mother-infant pairs attending the MCH clinic at six weeks (RR: 1.34, 95% CI: 1.07–1.68) and at 10 weeks postpartum (RR: 1.86, 95% CI: 1.34–2.58) [20]. A likely reason for which the retention rates in the study by Kebaya *et al*. are significantly higher than those by Odeny *et al*. is that the former assessed retention between delivery and six/ten weeks postpartum, while Odeny *et al*. measured retention during the longer time period between (antepartum) enrolment and eight weeks postpartum. Schwartz *et al*., an observational study judged to provide a very low quality of evidence, did not find any change in retention in ART at 12 months postpartum due to text messaging and phone calls (RR: 1.03, 95% CI: 0.83–1.27) [21]. Regarding integration of ANC and ART care, van Lettow *et al*. found that facilities, which require a referral between ANC and ART for all doses of ART had a higher rate of retention of women at 12 months after ART initiation than facilities where either the first or all doses of ART were provided in the ANC facility [23] (Table 8). Similarly, Weigel *et al*. reported that a series of more than 10 interventions over three years to improve the transition from ANC to ART was associated with higher retention in HIV care six months after ART initiation (RR: 3.85, 95% CI: 2.10–7.08) [24]. However, Turan *et al*., the only included randomized trial of an integration of care intervention, and Stinson *et al*. identified no effect of integration of care on postpartum retention (Tables 7–9) [22,25].

**Table 7:** Results of included studies – phone calls and/or text messages

Study	Outcome measure	Intervention group (%)	Control group (%)	Effect size	95% CI	*p*	Interpretation
Odeny *et al*., Kenya, randomized trial [19]	Percentage enrolled in PMTCT who attended a postpartum visit within eight weeks of giving birth	19.6	11.8	RR 1.66	RR 1.02–2.70	0.04	Two-way text messaging was associated with an increase in the proportion of patients who attended a postpartum visit within eight weeks of delivery
Kebaya *et al*., Kenya, randomized trial [20]	Percentage of MIPs who attended the Maternal and Child Health visit at six weeks postpartum	78.7	58.7	RR 1.34^a^	RR 1.07–1.68^a^	0.009	Biweekly phone calls during the first 10 weeks postpartum were associated with a higher proportion of MIPs attending the
	Percentage of MIPs who attended the Maternal and Child Health visit at 10 weeks postpartum	69.3	37.3	RR 1.86^a^	RR 1.34–2.58^a^	<0.0001	Maternal and Child Health clinic at six and 10 weeks postpartum
Schwartz *et al*., South Africa, cohort study [21]	Percentage retained at 12 months post-partum	78	76	RR 1.03^a^	RR 0.83–1.27^a^	0.81^a^	The use of text messages and phone calls was not associated with increased postpartum retention

CI=Confidence Interval; ART=Antiretroviral Therapy; RR=Relative Risk; MIP=Mother-infant pair.

a These values are not given in the original manuscript and were instead calculated by the authors of this review.

**Table 8:** Results of included studies – integration of care

Study	Outcome measure	Intervention group	Control group	Effect size	95% CI	*p*	Interpretation
Turan *et al*., Kenya, randomized trial [22]	Percentage enrolled in HIV care within 12 months after testing HIV+ in ANC^a^	69%	36%	OR 3.94	OR 1.14–13.63	—	Although integration of care was associated with increased and timelier ART initiation, it was not associated with a change in postpartum retention in HIV care
	Time from testing HIV+ in ANC to women's enrolment in HIV care (median days, IQR)	0 days (0–0)	8 days (0–72)	OR 2.20	OR 1.62–3.01	—	
	Percentage of eligible women who initiated ART within 12 months of testing HIV+ in ANC	40%	17%	OR 3.22	OR 1.81–5.72	—	
	Of those who enrolled in HIV care, the percentage of women with at least two HIV care follow-up visits in the first six months after testing HIV+ in ANC	48%	56%	OR 0.73	OR 0.47–1.14	—	
van Lettow *et al*., Malawi, cohort study [23]	% of HIV+ women (not already on ART) initiated on ART during pregnancy by facility	Model A: 82% Model B: 81% Model C: 80% Model D: N/A	N/A	—	Model A: 76%–87% Model B: 74%–89% Model C: 68%–91% Model D: N/A	0.96	ANC facilities requiring a referral to an ART clinic for the first and all subsequent doses of ART (Models C and D) were associated with a higher retention at 12 months after ART initiation than more integrated facilities (Models A and B)
	Likelihood of a facility in each model of care to have a retention rate >92% at six months after ART initiation relative to a facility in Model B (multivariable subgroup analysis)^b^	—	N/A	Model A: aOR 3.0 Model B: − Model C: aOR 5.4 Model D: aOR 9.1	A: aOR 0.7–12 B: − C: aOR 1.2–28 D: aOR 0.9–84	A: 0.1 B: − C: 0.04 D: 0.06	
	% retained at 12 months after ART initiation by facility^c^	Model A: 80% Model B: 77% Model C: 87% Model D: 95%	N/A	—	Model A: 77%–83% Model B: 73%–82% Model C: 83%–92% Model D: 88%–99%	0.002	
Weigel *et al*., Malawi, cohort study [24]	% retained at six months after ART initiation	2009: 65%	2006: 17%	RR 3.85^d^	RR 2.10–7.08^d^	<0.001	A series of interventions over three years to enhance linkage between ANC and ART was associated with much higher retention in ART care six months after ART initiation
Stinson *et al*., South Africa, cohort study [25]	% of those eligible for ART who initiated ART before delivery	Integrated model: 55% Proximal model: 48% Distal model: 47%	N/A	Proximal vs. integrated model: RR 0.88^d^ Distal vs. integrated model: RR 0.85^d^ Distal vs. proximal model: RR 0.97^d^	RR 0.72–1.07^d^ RR 0.69–1.06^d^ RR 0.76–1.24^d^	0.29	Three varying levels of ANC and ART integration were not associated with a different proportion of ART initiation among ART-eligible women by two years postpartum
	% of those eligible for ART who initiated ART within two years postpartum	Integrated model: 64% Proximal model: 67% Distal model: 55%^d^	N/A	Proximal vs. integrated model: RR 1.04^d^ Distal vs. integrated model: RR 0.85^d^ Distal vs. proximal model: RR 0.82^d^	RR 0.90–1.20^d^ RR 0.71–1.02^d^ RR 0.68–0.99^d^	0.08^d^	
	Of those who did not initiate ART before delivery, % who initiated ART within two years postpartum	Integrated model: 21% Proximal model: 35% Distal model: 14%	N/A	Proximal vs. integrated model: RR 1.58^d^ Distal vs. integrated model: RR 0.65^d^ Distal vs. proximal model: RR 0.41^d^	RR 1.00–2.52^d^ RR 0.33–1.28^d^ RR 0.22–0.78^d^	0.01	

CI=confidence interval; ART=antiretroviral therapy; RR=relative risk; OR=odds ratio; aOR=adjusted odds ratio.

a It was recommended to all HIV+ study participants to enrol in HIV care regardless of ART-eligibility.

b Variables that were included in the multivariate regression model are district in which the facility is located, facility type (district hospital, community hospital, health centre, private clinic), whether ART/PMTCT services are offered on all weekdays or only on certain weekdays, number of women in the study cohort at each facility, number of women in the study cohort per clinical staff, time of adherence counselling (on the day of ART initiation, at the next visit, both on the same as ART initiation and at the next visit), and availability of ART/mother-infant-pair clinic for follow-up.

c The denominator is all women not known to have transferred out.

d These values are not given in the original manuscript and were instead calculated by the authors of this review.

**Table 9:** Results of included studies – other studies

Author and year	Outcome measure	Intervention group	Control group	Effect size	95% CI	*p*	Interpretation
Kiweewa *et al*., Uganda, randomized trial [26]	Percentage who attended all scheduled visits	100%	98%	RR 1.02^a^	RR 0.98–1.07^a^	0.34^a^	Task-shifting to nurses along with home visits of defaulted patients by peer counsellors was not associated with a change in ART retention in the first 12 months after ART initiation
Mushamiri *et al*., Kenya, cohort study [27]	Percentage of women who attended six or more baby follow-up visits in the first 18 months postpartum	Area 1: 75%^a^ Area 2: 91%^a^ Area 1 & 2: 81%^a^	Area 3: 87%^a^	1 vs. 3: RR 0.86^a^ 2 vs. 3: RR 1.04^a^ 2 vs. 1: RR 1.21^a^ (1&2) vs. 3: RR 0.92^a^	RR 0.73–1.01^a^ RR 0.91–1.20^a^ RR 1.04–1.42^a^ RR 0.80–1.06^a^	0.04^a^	Within the CHW areas, the text messaging reminders were associated with a higher proportion of women attending six or more baby follow-up visits. Regardless of text messaging reminders, CHW services were not associated with a higher proportion of women attending six or more baby follow-up visits
Williams *et al*., UK, cohort study [28]	% of women who discontinued ART after delivery despite CD4 < 350	3.8%	4.0%	RR 0.96^a^	RR 0.06–14.55^a^	0.98	In the first 12 months postpartum financial support for formula feeding was associated with (1) fewer women on ART having a viral load ≥200, and (2) a lower mean number of missed ART appointments
	% of women with viral load ≥200 at any ART visit in first 12 months postpartum	20.0%	53.3%	RR 0.38^a^	RR 0.14–0.98^a^	0.04^a^
	Mean no. of ART appointments not attended in the first 12 months postpartum	1.73	3.08	—	—	<0.05
	% of MIPs who attended the Maternal and Child Health visit at 10 weeks postpartum	69.3%	37.3%	RR 1.86^a^	RR 1.34–2.58^a^	<0.0001

CI=confidence interval; ART=antiretroviral therapy; RR=relative risk; MIP=mother-infant pair.

a These values are not given in the original manuscript and were instead calculated by the authors of this review.

### Currently ongoing studies

Table 10 provides an overview of ongoing trials identified in our search. This list is likely to be non-exhaustive as it is restricted to studies that published a protocol in one of the databases searched for this review, which did not include trial registers. On this note, we would also like to point readers to the randomized trial by Yotebieng *et al*. [29], which was published after this systematic review had been completed. Eight of the studies in Table 10 are cluster-randomized trials and one is a stepped wedge randomized trial. Four studies are evaluating an intervention that employs peer counsellors, two trials examine the effect of integration of HIV and non-HIV clinical services in the same clinic, and two studies’ interventions include male participation in PMTCT. Seven studies assess retention in the first 12 months postpartum, and one study follows women for two years after birth. Two studies only assess short-term retention in MCH care (at six and 12 weeks postpartum).

**Table 10:** Non-exhaustive list of ongoing studies evaluating interventions to improve postpartum PMTCT or ART retention

Author and year					Sample size				
							
	Country	Study period	Study design	Study population	*Intervention*	*Control*	Intervention	Control group	Relevant outcome measure
Baernighausen 2015 [30]	South Africa	July 2015 to January 2017	Stepped wedge randomized trial	Pregnant women aged ≥18 years	Seven facilities (3000 women)	Seven facilities (3000 women)	Quality improvement of clinic processes for maternal and child health	Standard of care	Attendance of MCH visit at six weeks postpartum
Jones *et al*. 2014 [31]	South Africa	April 2014 to June 2018	2×2 cluster-randomized trial	HIV+ women at 8–24 weeks gestation who have a male partner	Six facilities (720 women)	Six facilities (720 women)	(1) Male participation in PMTCT (2) Group and individual counselling sessions	(1) No male participation in PMTCT (2) No group and counselling sessions	Attendance of PMTCT care at six and 12 months postpartum
Foster *et al*. 2014 [32]	Zimbabwe	July 2014 to December 2016	Cluster-randomized trial	HIV+ women ≤34 weeks gestation	15 facilities (≥150 women)	15 facilities (≥150 women)	Availability of a mother-support group for HIV+ women at the facility	No availability of a mother-support group for HIV+ women at the facility	Attendance of postpartum ART visits at least once every two months during the first 12 months postpartum
Sam-Agudu *et al*. 2014 [33]	Nigeria	April 2014 to September 2016	Cohort study	HIV+ women	Ten facilities (240 MIPs)	Ten facilities (240 MIPs)	Support by mentor mothers	Standard of care (informal peer support)	Retention of MIPs in PMTCT at six and 12 months postpartum
Rosenberg *et al*. 2014 [34]	Malawi	November 2013 to July 2016	Three-arm cluster-randomized trial	Pregnant and breastfeeding women with a new diagnosis of HIV at ANC	Arm 1: ≥360 women Arm 2: ≥360 women	Arm 3: ≥360 women	Arm 1: Facility-based peer support plus follow-up if a clinic appt is missed Arm 2: Community-based peer support plus follow-up if a clinic appt is missed	Arm 3: Standard of care (routine facility-based adherence counselling)	Proportion of women retained (no ≥60 day period without ART) in Option B+ until two years postpartum
Oyeledun *et al*. 2014 [35]	Nigeria	May 2014 to February 2016	Cluster-randomized trial	HIV+ women ≤34 weeks gestation, ART-naïve and plan to take ART for ≥6 months postpartum	16 facilities (320 women)	16 facilities (320 women)	Quality improvement intervention using a Breakthrough Series	Standard of care	Proportion of ART visits attended in the first 12 months postpartum
Mangwiro *et al*. 2014 [36]	Zimbabwe	January 2014 to January 2016	Cluster-randomized trial	HIV+ women ≤38 weeks gestation	16 facilities (416 women)	16 facilities (416 women)	Provision of point-of-care CD4 testing	Standard of care (laboratory-based CD4 testing)	Proportion of scheduled ART visits attended through the first 12 months on ART
Mwapasa *et al*. 2014 [37]	Malawi	May 2013 to [end date not given]	Three-arm cluster-randomized trial	HIV+ pregnant women	Arm 1: Ten facilities (410 MIPs) Arm 2: 10 facilities (410 MIPs)	Arm 3: Ten facilities (410 MIPs)	(1) Arm 1: Provision of HIV and non-HIV services in same clinic (integrated care) (2) Arm 2: Integrated care plus SMS-based (instead of paper-based) notification of community-based volunteers for tracing of defaulters at home	Standard of care	Proportion of scheduled PMTCT/ART visits attended in the first 12 months postpartum
Aliyu *et al*. 2013 [38]	Nigeria	Two years [no start and end date given]	Cluster-randomized trial	HIV+ pregnant women not on ARVs at the first ANC visit or at delivery	Six facilities (150 women)	Six facilities (150 women)	Task-shifting from physicians to nurses, midwives and CHWs *plus* PoC CD4 testing *plus* integration of MCH and ART visits postpartum *plus* written invitations to male partners to attend PMTCT	Standard of care	Retention of MIPs at 12 weeks postpartum
Rotheram-Borus *et al*. 2011 [39]	South Africa	[no start and end date given]	Cluster-randomized trial	HIV+ women <34 weeks gestation and enrolled in PMTCT	Four facilities (600 women)	Four facilities (600 women)	Four antenatal and Four postnatal small group sessions led by a peer counsellor	Standard of care	Clinic attendance at six and 12 months postpartum

MCH=maternal and child health; HIV+=HIV-positive; ART=antiretroviral therapy; ANC=antenatal care; PMTCT=prevention of mother-to-child HIV transmission; Appt=appointment; MIP=mother-infant pair; ARVs=antiretroviral drugs; CHWs=community health workers; PoC=point of care; CD4=cluster of differentiation 4 cell count.

### Summary of the evidence

This review has identified 10 studies that met the inclusion criteria, of which we only judged four to be of high or moderate quality evidence. Two randomized trials found that the group receiving text messaging and/or phone calls had higher postpartum retention. Unfortunately, neither study assessed long-term retention. Odeny *et al*. found that two-way text messaging led to an increase in attendance of a postpartum visit within eight weeks of delivery [19], and Kebaya *et al*. found that women living with HIV who received biweekly phone call reminders during the postpartum period were more likely to attend the baby follow-up visit at six weeks and at 10 weeks postpartum [20]. Based on this, we feel that phone-based interventions seem to hold promise in increasing retention in the early postpartum period. Only one of the currently ongoing trials we identified is evaluating a phone-based intervention [34]. It is important to note that the retention rate in the control group in both included trials was low (11.8% at eight weeks postpartum in Odeny *et al*. [19], and 37.3% at 10 weeks postpartum in Kebaya *et al*. [20]), which may limit the generalizability of the findings to settings with higher retention and could (partially) explain the lack of effect found by Schwartz *et al*. [21].

Integration of care may have a variety of benefits, such as higher ART initiation of pregnant women living with HIV; this review, however, focused only on the effect of care integration on postpartum ART retention. The evidence for the impact of integration of care interventions on postpartum ART transition and retention is inconsistent, which is likely due to the heterogeneity of intervention designs, outcome measures, and study populations, as well as the low study quality of the four identified studies. In particular, each of the studies integrated care between ANC and ART services somewhat differently, making the evidence arising from these studies highly context-specific and difficult to generalize to other settings. In addition, only one of the studies [25] included a completely integrated model of care whereby the ANC clinic provides ART to the pregnant women for life, obviating the need for referral to an ART clinic (a care transition that is likely accompanied by a high loss to follow-up) either during pregnancy or in the postpartum period. Only one of the four studies found that integration of care improved retention in HIV care postpartum [24]. However, although the study found a stark increase in the percentage of women retained at six months after ART initiation (65% vs. 17%; RR 3.85, 95% CI: 2.10–7.08) [24], it suffers from a high risk of bias from the pre-post study design with a large time gap (three years) between the pre- and post-data collection periods. In addition, the study implemented 11 changes aimed at improving ANC to ART linkage during the study period, which makes it impossible to determine which of the measures was effective. The only randomized trial on this topic, a cluster-randomized trial conducted by Turan *et al*. in Kenya [22], found no difference in the proportion of women with two or more HIV care follow-up visits in the first six months after testing HIV-positive in ANC between integrated clinics (providing ANC and ART services in the same clinic until 18 months postpartum) and non-integrated clinics (ANC clinics that referred pregnant women for ART). The study, however, was insufficiently powered for this outcome. Nonetheless, the trial found that integration of care can lead to improved transitioning between PMTCT and ART as measured by the higher percentage of ART-eligible women who initiated ART within 12 months of testing HIV-positive in ANC in integrated as compared to non-integrated care clinics (40% vs. 17%; OR 3.22; 95% CI: 1.81–5.72).

The third randomized trial included in this review, a non-inferiority study, found that task-shifting to nurses along with home visits of defaulted patients by peer counsellors did not lead to a change in ART retention in the first 12 months after ART initiation [26]. The potential for the study's intervention programme to have a beneficial effect on retention was limited by the extremely high retention rate in the control group (98%). The only study not carried out in sub-Saharan Africa found that providing financial support for formula feeding to women at certain postpartum clinic visits increased both ART retention and adherence as measured by viral suppression [28]. However, the study, which was carried out in the United Kingdom, has limited generalizability to countries, in which exclusive breastfeeding is the recommended infant feeding option for pregnant women living with HIV. In addition, the financial support provided (a 2015 equivalent of US$930 per woman) is well above what health systems in low-and middle-income countries can currently afford, even if this value is adjusted for purchasing-power-parity.

Concerning the use of monetary incentives to increase postpartum ART retention, we would like to point out that a large randomized trial, published after we had completed this systematic review, evaluated the effect of conditional cash transfers on retention in PMTCT at six weeks postpartum in Kinshasa [29]. The trial found a 12.5% higher probability of retention at six weeks postpartum for women in the intervention compared to the control arm (81% vs. 72%; RR ratio: 1.11; 95% CI: 1.00–1.24), which was marginally significant (*p*=0.055). The monetary incentive used in this trial (US$5 at the first visit plus US$1 at every subsequent PMTCT visit conditional on attending the scheduled visit and on a few other requirements, such as providing a blood sample for CD4-cell count measurements) is far smaller than the one used in the study in the United Kingdom but may still be considered to be too high for scale-up in many settings in sub-Saharan Africa. Feasibility concerns aside, it is important to consider this cash value in the context of substantial out-of-pocket expenditures and opportunity costs incurred by patients who attend HIV care in sub-Saharan Africa, despite ART being free at the point of care [40].

The evidence on approaches to improve the transition between MCH and long-term ART services and retention of women in ART programmes postpartum is currently still very limited, even though a number of relevant randomized trials are currently under way in sub-Saharan Africa. Table 10 provides a non-exhaustive list of currently ongoing trials. However, most of these trials will not yield final results before 2016. This recent surge in research interest on the question of postpartum ART transitioning and retention most likely reflects the policy shift to lifelong ART for pregnant women. The fairly rapid move to Option B+ by many countries over the last few years may partially explain the current lag in research evidence on some of the operational questions of implementing lifelong ART for all pregnant and breastfeeding women living with HIV.

Unfortunately, only one of the ongoing trials listed in Table 10 assesses retention beyond 12 months postpartum [34]. It is, however, crucial to assess longer term postpartum retention on ART because (1) a major anticipated benefit of lifelong ART is that HIV-positive women will be virally suppressed at the time of subsequent pregnancies, which requires sustained ART adherence; (2) the duration of breastfeeding, a period of high risk of vertical HIV transmission [41], tends to be considerably longer than 12 months in sub-Saharan Africa [42]; and (3) the transition between maternal health services to general ART care, which may occur after 12 months postpartum depending on the country setting, is probably a point in the care cascade at which women are at a particularly high risk of being lost from care. We, therefore, anticipate that the research gap on long-term postpartum ART retention will remain in the coming years.

### Learning from research on adult ART retention, adherence, and linkage to HIV care

While the evidence base on the retention of women specifically in the postpartum period is limited, it is plausible that interventions increasing ART retention in general also improve postpartum ART retention. Thus, valuable lessons could be learned from studies aimed at improving: (1) retention and adherence in ART programmes, or (2) linkage between different HIV care settings, such as from HIV testing to pre-ART or ART initiation, or from pre-ART care to ART. Regarding adherence, Chaiyachati *et al*. found in their rapid systematic review that five broad types of interventions, namely cognitive behavioural therapy, education, treatment supporters, directly observed therapy, and active reminder devices (e.g., text messaging), were effective in several settings in improving adherence to ART [43]. Concerning linkage between HIV care settings, a systematic review by Fox *et al*. identified several intervention types that proved to be effective in improving linkage between HIV testing and enrolment in HIV care with technology-based interventions, such as text messaging and point-of-care CD4-testing, appearing to hold the greatest promise (study under peer review). Point-of-care CD4 testing is, of course, less likely to have a substantial effect for linking pregnant women to ART as they are eligible for ART (or Zidovudine) regardless of CD4-cell count. Another recent systematic review found that food incentives and point-of-care CD4 testing increased the percentage of eligible adults who initiated ART [44]. Thus, although the evidence base for the effectiveness of phone-based interventions in improving postpartum retention comes from only two studies, there is a broader body of evidence that phone-based interventions can increase adult ART retention, and linkage between HIV testing and HIV care.

### Learning from non-intervention studies on postpartum retention

An additional important source of evidence that can inform the design of interventions aimed at improving postpartum ART retention is non-intervention studies, usually cohort studies, on factors associated with non-retention in HIV care. Even though some factors associated with non-retention in ART care in general are likely to also apply to pregnant and postpartum women, it is likely that pregnant women face additional barriers specific to their gender and pregnancy status. In a recent systematic review, Hodgson *et al*. identified a variety of individual and community-level factors associated with non-retention in HIV care among pregnant and postpartum women, including poor understanding of HIV and its treatment, non-disclosure of HIV status to a spouse, and partner involvement in PMTCT [46]. At the community-level, HIV-related stigma was still a major barrier to retention. In addition, Colvin *et al*. identified a number of health system barriers to HIV care retention of pregnant and postpartum women, including insufficient communication and coordination between different health system actors, poor training of healthcare providers, and a stigmatizing attitude by some healthcare workers [47].

These reviews clearly show that the reasons for non-retention of pregnant and postpartum women on ART are complex and multifaceted, including patient-level factors (knowledge and conflicting priorities, such as caring for family and employment), community-level factors (such as stigma), provider-level factors (such as clinical knowledge and attitudes), and wider health system barriers (such as drug stock outs, access to care, and waiting times). This might suggest that multifaceted interventions that attempt to address as many of these barriers as possible will be most effective in improving postpartum ART retention. However, these interventions also tend to be more costly and complex to implement. While the effect sizes are modest, the two randomized trials on phone calls and/or text messages included in this review showed that simple singular interventions can significantly improve postpartum ART retention [19,20]. Thus, even though multifaceted interventions may be needed to achieve near-perfect retention, it is unclear whether simple singular interventions or more complex multifaceted interventions will prove to be more cost-effective in improving postpartum retention in health systems that currently experience a high rate of loss to follow-up of postpartum women living with HIV. It is, therefore, essential that studies on this topic assess and report the cost of implementing the intervention that is being evaluated. However, the utilitarian cost-effectiveness criterion should not be the only lens through which these interventions are evaluated. After all, stigma towards people living with HIV both by community members and healthcare providers has important human rights implications, which can justify interventions independently of cost-effectiveness considerations.

### Limitations

This systematic review has several limitations. First, we may not have identified all studies that have been conducted on this topic as the search strategy was only run in English and we were, therefore, not able to identify studies without an English title or abstract. In addition, although we made an effort to identify grey literature by contacting corresponding authors of the included studies and study protocols, we may not have identified unpublished data on our review questions. Second, due to the wide variation in interventions, study designs, and outcome measures, it was not feasible to summarize the results of the included studies using Forest plots or a meta-analysis. Third, the small number and generally poor quality of the identified studies limited our ability to deduce meaningful conclusions with policy implications.

## Conclusions

The evidence base on interventions to improve retention of women in HIV care during the postpartum period is weak, particularly for improving longer term retention on ART. Nevertheless, there is some evidence that phone-based interventions can improve retention in PMTCT in the first one to three months after childbirth. A number of randomized trials are currently under way and expected to publish their results in mid- to late 2016. Our systematic review has identified a weak evidence base on key operational aspects of implementing the WHO's recommendation of lifelong ART for all pregnant and breastfeeding women living with HIV. This study, therefore, highlights the need for more rigorous evaluations of health system interventions to determine the most efficient and effective strategies of providing ART to this important population group.
